# Epidemiological Study of RRT-Treated ESRD in Nanjing - A Ten-Year Experience in Nearly Three Million Insurance Covered Population

**DOI:** 10.1371/journal.pone.0149038

**Published:** 2016-02-18

**Authors:** Yu-Chen Han, Han-Ming Huang, Ling Sun, Chao-Ming Tan, Min Gao, Hong Liu, Ri-Ning Tang, Yan-Li Wang, Bei Wang, Kun-Ling Ma, Bi-Cheng Liu

**Affiliations:** 1 Institute of Nephrology, Zhongda Hospital, Southeast University School of Medicine, Nanjing, Jiangsu, China; 2 Social Insurance Management Center, Nanjing Municipal Human Resources and Social Security Bureau, Nanjing, Jiangsu, China; 3 Southeast University School of Public Health, Nanjing, Jiangsu, China; Mario Negri Institute for Pharmacological Research and Azienda Ospedaliera Ospedali Riuniti di Bergamo, ITALY

## Abstract

**Background:**

The growing burden of end-stage renal disease (ESRD) has been a great challenge to the health care system of China. However, the exact epidemiological data for ESRD in China remain unclear. We aimed to investigate the epidemiology of ESRD treated by renal replacement therapy (RRT) in Nanjing based on analysing ten-year data of Nanjing three million insurance covered population.

**Methods:**

Using the electronic registry system of Urban Employee Basic Medical Insurance (UEBMI), we included all subjects insured by UEBMI in Nanjing from 2005 to 2014 and identified subjects who developed ESRD and started RRT in this cohort.

**Results:**

The UEBMI population in Nanjing increased from 1,301,882 in 2005 to 2,921,065 in 2014, among which a total of 5,840 subjects developed ESRD and received RRT. Over the 10-year period, the adjusted incidence rates of RRT in the UEBMI cohort gradually decreased from 289.3pmp in 2005 to 218.8pmp in 2014. However, the adjusted prevalence rate increased steadily from 891.7pmp in 2005 to 1,228.6pmp in 2014. The adjusted annual mortality rate decreased from 138.4 per 1000 patient-years in 2005 to 97.8 per 1000 patient-years in 2014. The long-term survival rate fluctuated over the past decade, with the 1-year survival rate ranging from 85.1% to 91.7%, the 3-year survival rate from 69.9% to 78.3% and the 5-year survival rate from 58% to 65.4%.

**Conclusion:**

Nanjing is facing an increasing burden of ESRD with its improvement of medical reform. The ten-year complete registry data on RRT in urban employees in Nanjing provided a unique opportunity to understand the real threat of ESRD confronting China during its process of health care transition.

## Introduction

China experienced an unprecedented process of rapid urbanization over the past decade, which reshaped the nation’s health profile with an increasing burden of non-communicable disease (NCD) [1–3]. End-stage renal disease (ESRD) is only one of the emerging burdens of NCD, challenging the health care system to provide renal replacement therapy (RRT) to this increasing number of patients [4]. Over the past decade, with its economic success, the Chinese government has taken great efforts to improve the insurance reimbursement policy for patients with ESRD, greatly increasing the growth rate of patients with RRT. However, due to the lack of the accurate national registry system, the exact epidemiological data for ESRD in China remain unclear.

In 2000, the government of Nanjing (the capital city of Jiangsu Province, located in the southeast of China) established an outpatient reimbursement program for RRT (as a part of the Urban Employee Basic Medical Insurance, UEBMI) for all insured urban employees who suffered from ESRD [5]. This reimbursement program of UEBMI covers 90% of the RRT related expenditure, affording all insured ESRD patients access to RRT [5]. At the same time, the city government introduced a complete electronic registry system, requesting that all insured patients be recorded at each session of RRT. Now, fifteen years have passed, almost three million members of the UEBMI-covered population provided us a unique data system to understand the real world of ESRD in the Chinese population.

In this study, we investigated the prevalence and incidence rate of RRT using the UEBMI electronic registry system. For the first time, we provided the epidemiological data of nearly three million members of the Chinese urban population from the last ten years, which we believe to be a reliable window for us to learn about the real burden of ESRD in China.

## Subjects and Methods

### RRT reimbursement program of UEBMI

The UEBMI is a mandatory social insurance for urban employee in China. The employee of all the urban enterprises, individual economic organizations, private non-enterprise organizations and social groups (including the retiree) and those with liberal professions in the urban district should be enrolled in the UEBMI according to the Labour Law of the People’s Republic of China. Urban residents without employment are covered by Urban Resident Basic Medical Insurance (URBMI) and rural residents are covered by New Cooperative Medical Scheme (NCMS). These two insurance are not mandatory, but the insured are subsidized by the government. In 2010, the coverage of the target population for UEBMI, URBMI and NCMS was 92.4%, 92.9% and 96.6%, respectively, in China.

For the insured urban employee who developed ESRD, the government of Nanjing established an outpatient reimbursement program for RRT in 2000 as an important supplemental part of UEBMI. The ESRD patients in UEBMI should be diagnosed by senior nephrologists from tertiary hospitals and their need for RRT be assessed according to the criteria (major criteria: for established CKD patients without diabetes, when eGFR decreases to less than 10ml/min/1.73m^2^; for CKD patients with diabetes, when eGFR decreases to less than 15ml/min/1.73m^2^) before they could be enrolled in the program and be financially reimbursed for RRT. Once the eligibility is verified, their basic information and the sequential RRT information will be recorded in the electronic registry system. URBMI and NCMS also have their RRT reimbursement programs. However, these programs have a rather short history and lack a complete RRT registry system.

### Selection of subjects

Through the use of RRT registry system in UEBMI, we extracted the information of all the subjects registered for RRT in UEBMI cohort in Nanjing and 6,017subjects were collected. Among these patients, 109 subjects were excluded because of misclassification as RRT. We also excluded those deceased before 2005 (28 subjects) and those initiated RRT after 2014 (10 subjects). Among the selected RRT patients in UEBMI cohort (2005–2014), those registered for RRT reimbursement program but did not receive RRT (5 subjects) or those did not receive RRT in the region of Nanjing (8 subjects) or those with incomplete information (17 subjects) were excluded. For the included RRT patients (5840 subjects) in UEBMI cohort from 2005 to 2014, basic information such as gender, date of birth, date of death (for deceased subjects), date of RRT initiation and modality of RRT were collected for further analysis.

### Statistical analysis

For the included subjects registered for RRT in UEBMI cohort, we divided them into five age groups (18 to 44 years old, 45 to 54 years old, 55 to 64 years old, 65 to 74 years old and ≥ 75 years old). The UEBMI cohort was also divided in this way. The prevalence, incidence and annual mortality rate of RRT in UEBMI cohort for each age level each year were calculated. Annual rate for each gender were also calculated. For comparison between rates for UEBMI cohort in each year, UEBMI cohort in 2014 was used as the standard population. The percentage of the UEBMI subjects in each level (five age levels multiplied by two sex levels) for each other year from 2005 to 2013 was adjusted to the percentage of the UEBMI subjects in the same level in 2014. Overall rates are adjusted for age and sex. Rates by sex are adjusted for age. Rates by age are adjusted for sex. The changing patterns of the incidence and the mortality were similar, both consisting of two phases, namely the descending phase and the following plateau phase. Piecewise regression was used to model this pattern and to test the change of the slope of the two phases (S3 File). Unadjusted survival rates (1-year survival rates, 3-year survival rates, 5-year survival rates) are calculated using Kaplan-Meier methods for each incident cohort for each year. Survival rates are compared using Log-rank test. We performed all of statistical analyses with Microsoft Excel and Stata version 13.0 (Stata Corp, College Station, Tex., USA). Two-tailed *P* < 0.05 was considered as significant.

### Ethics

Our study was approved by the ethics committee of Zhongda Hospital, Southeast University (2015ZDSLYY035.0) and adhered to the 1964 Helsinki declaration and its later amendments or comparable ethical standards. Informed consent was waived by the ethics committee.

## Results

### The UEBMI cohort: 2005–2014

The UEBMI cohort consisted of all the urban employees in Nanjing, and it increased by 124% from 1,301,882 in 2005 to 2,921,065 in 2014. During the same period, the resident population of Nanjing increased only 8.9% from 5.96 million in 2005 to 6.48 million in 2014. The UEBMI cohort as a percentage of the resident population of Nanjing increased steadily from 22% in 2005 to 45% in 2014. During the ten-year period, the sex ratios of the UEBMI cohort were consistently around 106–108:100 (male: female). Subjects age 18–64 constituted the majority of the UEBMI cohort throughout the decade, increasing slightly from 85% in 2005 to 88% in 2014 (Table 1). Hemodialysis constituted the major modality of RRT in prevalent ESRD patients in UEBMI, rising slightly from 72% in 2005 to 77% in 2014, while peritoneal dialysis increased from 4% in 2005 to 10% in 2014, and kidney transplantation decreased from 24% in 2005 to 13% in 2014 (S1 Fig). As for the incident ESRD patients in UEBMI, a similar pattern of trend was noted (S2 Fig).

**Table 1:** The UEBMI cohort in Nanjing during the ten-year period (2005–2014).

	2005	2006	2007	2008	2009	2010	2011	2012	2013	2014
**Resident Population (1,000)**	5958.0	6072.3	6171.7	6244.6	6297.7	6324.2	6363.6	6384.7	6430.9	6487.2
**Sex Ratio (Female = 100)**	105.1	104.6	104.0	103.4	102.8	102.2	101.7	101.4	100.9	100.4
**15–64 yr**	NA	NA	NA	NA	NA	NA	79.5%	80.0%	80.4%	NA
**≥65yr**	NA	NA	NA	NA	NA	NA	9.2%	9.7%	9.9%	NA
**Urban Population (1,000)**	3790.9 (63.6)*	3960.5 (65.2)	4073.2 (66.0)	4161.2 (66.6)	4227.3 (67.1)	4252.5 (67.2)	4310.3 (67.7)	4355.1 (68.2)	4395.9 (68.4)	4452.0 (68.6)
**Rural Population (1,000)**	2167.1 (36.4)	2111.8 (34.8)	2098.5 (34.0)	2083.4 (33.4)	2070.4 (32.9)	2071.7 (32.8)	2053.3 (32.3)	2029.6 (31.8)	2035.0 (31.6)	2035.2 (31.4)
**UEBMI Population (1,000)**	1301.9	1447.2	1611.1	1900.3	2050.7	2225.2	2441.2	2577.0	2802.1	2921.1
UEBMI / Resident population(%)**	21.9	23.8	26.1	30.4	32.6	35.2	38.4	40.4	43.6	45.0
**Sex Ratio (Female = 100)**	108.4	107.4	106.9	107.4	108.1	107.6	106.9	106.5	106.2	106.4
**18–64 yr**	85.1%	85.8%	86.7%	88.0%	88.4%	88.8%	89.3%	89.2%	88.6%	88.2%
**≥65yr**	14.8%	14.1%	13.3%	11.9%	11.6%	11.1%	10.6%	10.8%	11.4%	11.7%
**ESRD Population**	1263	1493	1754	2006	2220	2457	2710	2984	3300	3588

UEBMI, Urban Employee Basic Medical Insurance; ESRD, End stage renal disease; HD, hemodialysis; PD, peritoneal dialysis; Tx, transplantation; NA, not available.

*Numbers in the brackets were presented as the percentage of the resident population;

** The percentage of the resident population in Nanjing covered by UEBMI.

### Incidence

**Fig 1:**
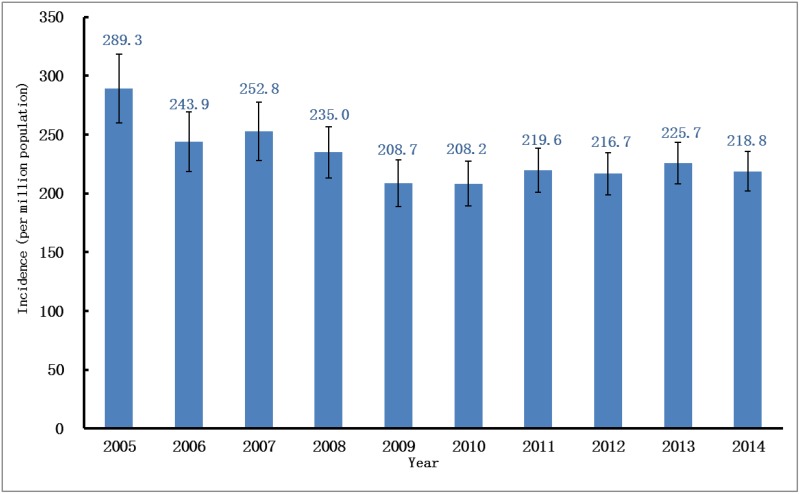
Incidence rate of RRT in UEBMI cohort. Incidence rates per million populations (adjusted for age and gender) over a ten-year period. RRT, renal replacement therapy; UEBMI, Urban Employee Basic Medical Insurance.

**Fig 2:**
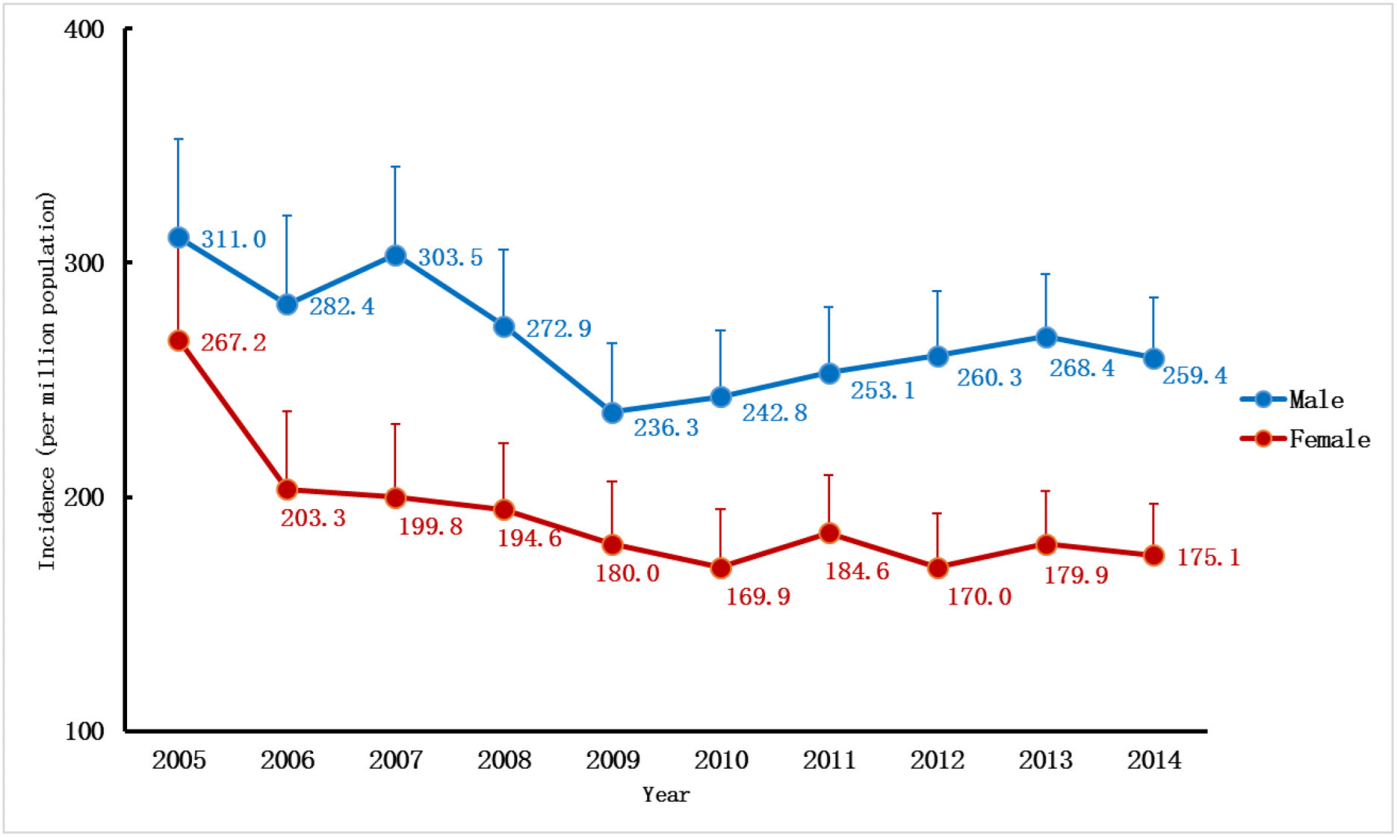
Gender-specific incidence rate of RRT in UEBMI cohort. Incidence rates per million populations by genders (adjusted for age) over a ten-year period. RRT, renal replacement therapy; UEBMI, Urban Employee Basic Medical Insurance.

**Fig 3:**
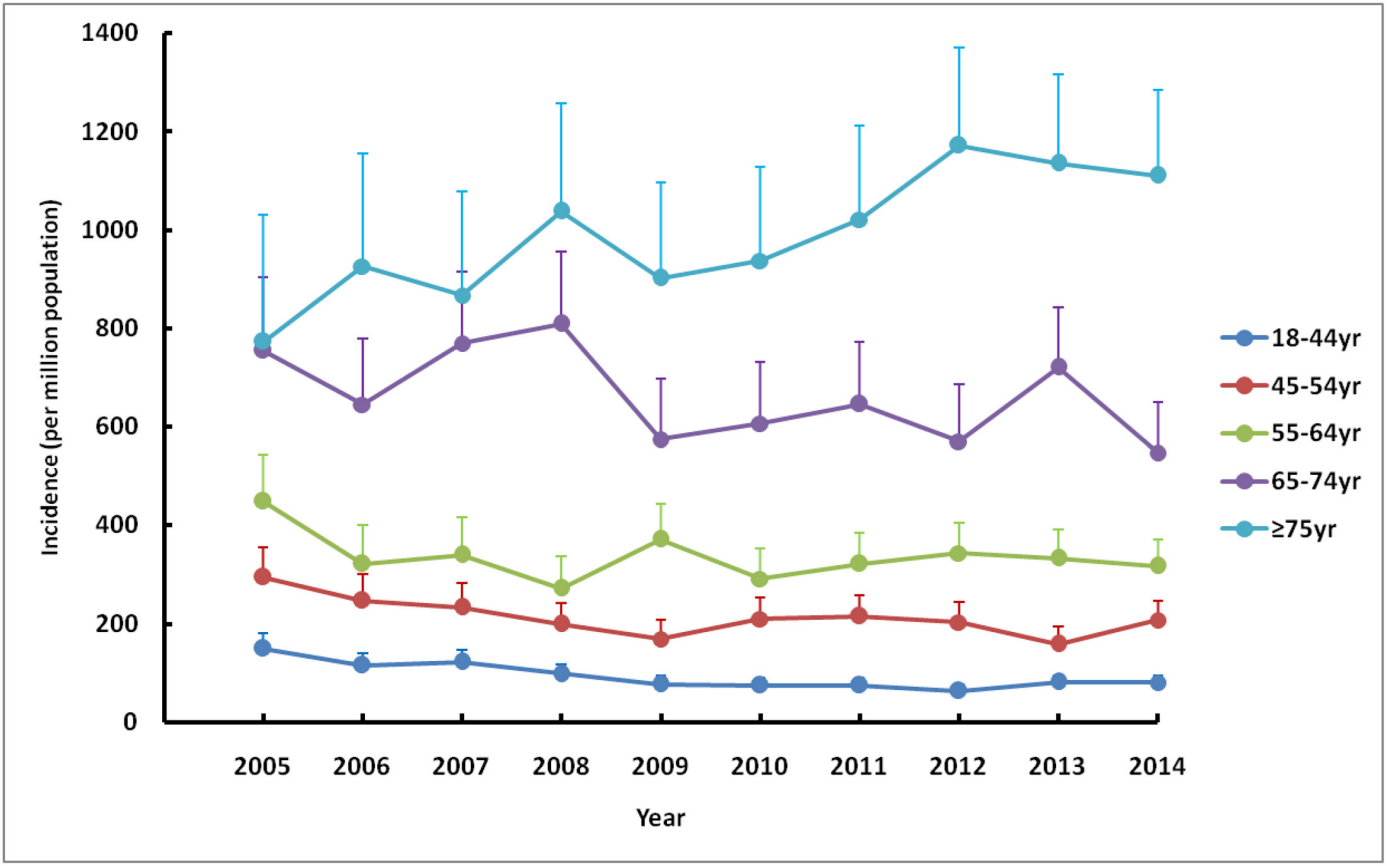
Age-specific incidence rate of RRT in UEBMI cohort. Incidence rates per million populations by selected age categories (adjusted for gender) over a ten-year period. RRT, renal replacement therapy; UEBMI, Urban Employee Basic Medical Insurance.

Over the 10-year period, the incidence trend could be divided into two parts: the descending phase, from 2005 to 2009; and the plateau phase, from 2010 to 2014. Over the first five years, the adjusted incidence rate of ESRD decreased by more than 20%, from 289.3pmp in 2005 to 208.7pmp in 2009. However, the descending trend virtually went into a plateau since 2010, with only a slight increase of 5% over the last five years, reaching an adjusted incidence rate of 218.8pmp in 2014 (Fig 1, S3 File). The patterns of the incidence trends were similar for both sexes, with male subjects having consistently higher adjusted rates than female subjects during the past decade (Fig 2). Regarding age-specific rates, the elderly had much higher adjusted rates than younger people. In 2014, the adjusted incidence rates increased significantly with the increase in patients’ ages; from 80.3pmp for those age 18–44 to 547.2pmp for those age 65–74 and surging to 1,110.4pmp for those age 75 and older. The patterns of the incidence trends were much different between these age groups. For those age 18–74, the adjusted incidence rates of ESRD all decreased since 2005, whereas rate for those age 75 and older increased by more than 40% from 772.9pmp in 2005 to 1,110.4pmp in 2014 (Fig 3).

### Prevalence

**Fig 4:**
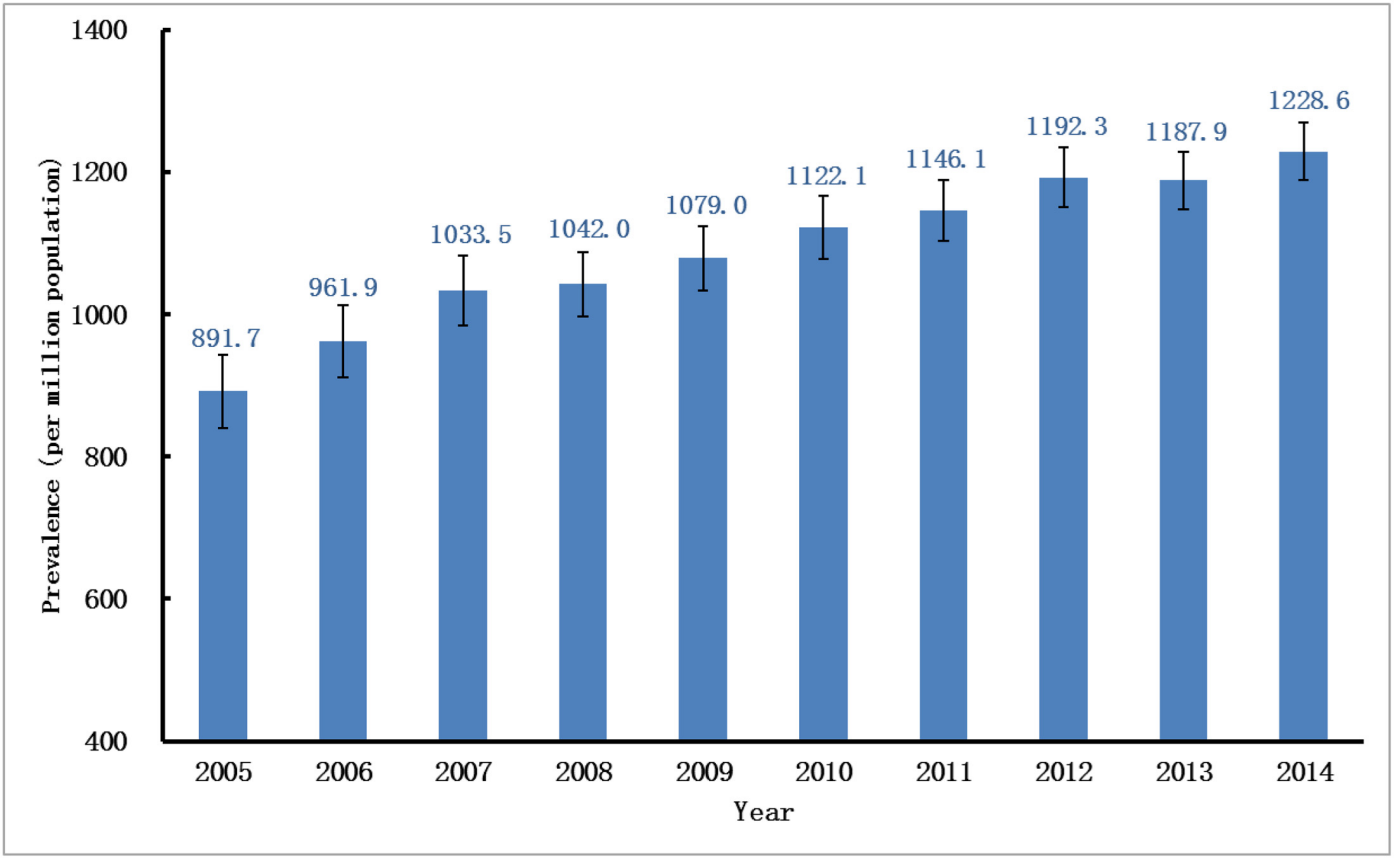
Prevalence rate of RRT in UEBMI cohort. Prevalence rates per million populations (adjusted for age and gender) over a ten-year period. RRT, renal replacement therapy; UEBMI, Urban Employee Basic Medical Insurance.

**Fig 5:**
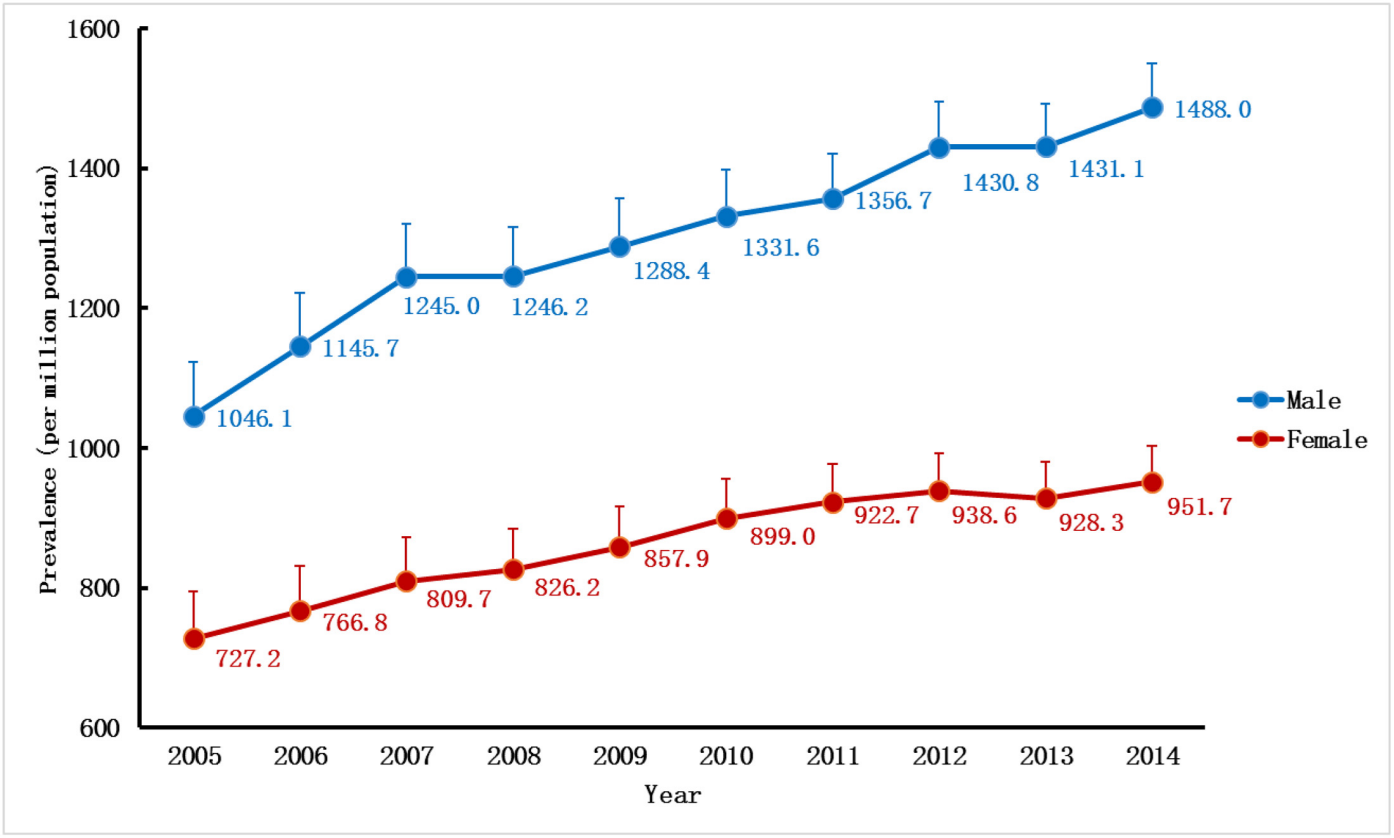
Gender-specific prevalence rate of RRT in UEBMI cohort. Prevalence rates per million populations by genders (adjusted for age) over a ten-year period. RRT, renal replacement therapy; UEBMI, Urban Employee Basic Medical Insurance.

**Fig 6:**
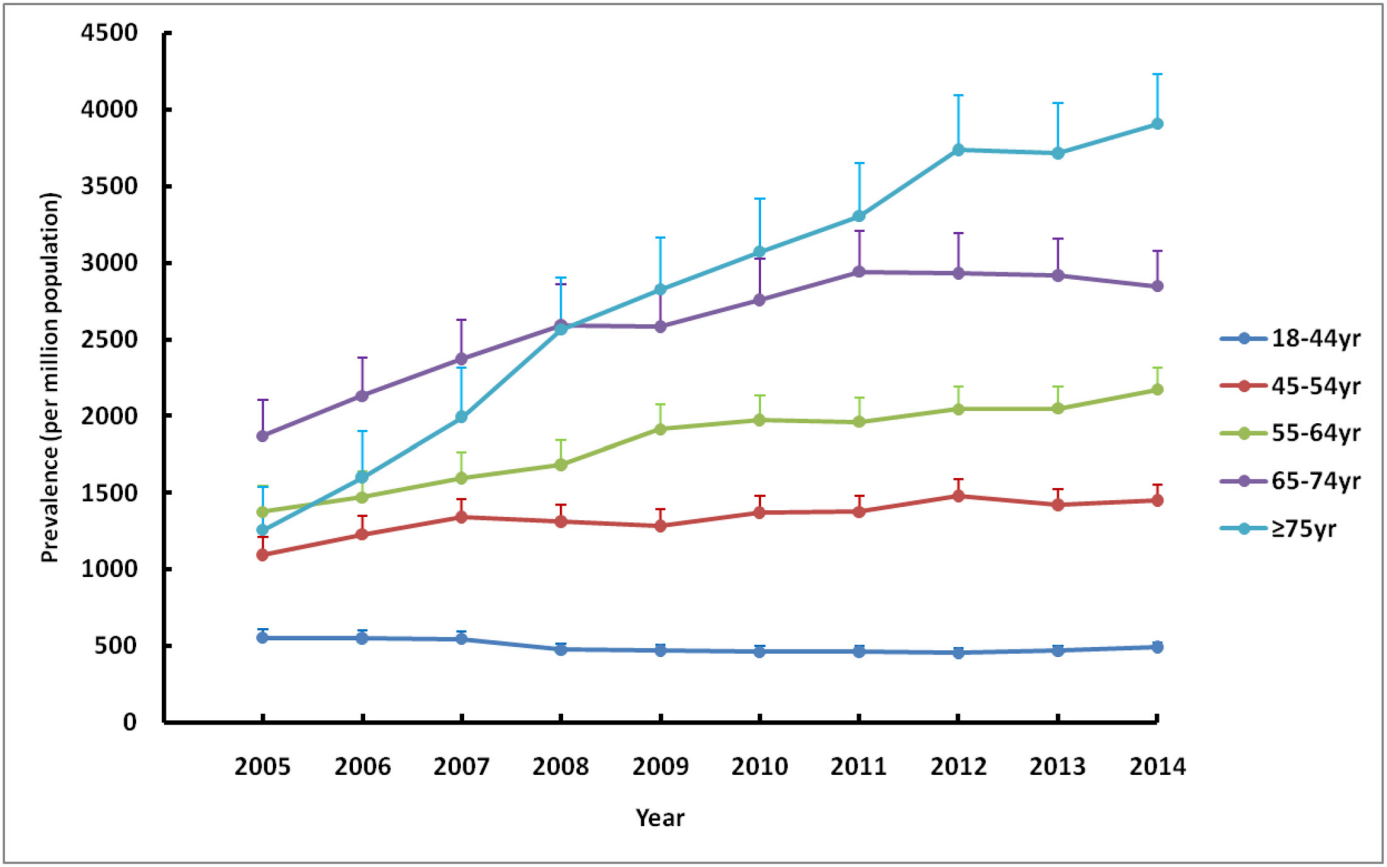
Age-specific prevalence rate of RRT in UEBMI cohort. Prevalence rates per million populations by selected age categories (adjusted for gender) over a ten-year period. RRT, renal replacement therapy; UEBMI, Urban Employee Basic Medical Insurance.

During the same period, the adjusted prevalence rates increased each year except for 2013, rising from 891.7pmp in 2005 to 1,228.6pmp in 2014 (Fig 4). The prevalence trend could also be divided into two parts. During the first phase before 2008, the annual growth rate was as high as 7%, whereas such rapid growth rates quickly slowed down in 2008 and fluctuated around 3% during subsequent years. Such growth patterns in the prevalence were similar for both sexes. Similarly, the adjusted prevalence rates of the males were consistently higher than those of the females throughout the decade (Fig 5). Regarding age-specific rates, the adjusted prevalence rates increased gradually with age, increasing by approximately 700–1,100pmp for every ten-year interval and rising from 490.5pmp for those age 18–44 to 3,904.7pmp for those age 75 and older (data in 2014). The patterns of the prevalence trends were also different among age groups. Over the ten-year period, the adjusted prevalence rate for those age 75 and older experienced a striking 210% increase, while for those age 45–74, the increases were much slower, with total increases ranging from 30% to 50%. In contrast, for adults younger than 45 years old, the adjusted prevalence rate decreased slightly by 10% during the same period (Fig 6).

### Mortality

**Fig 7:**
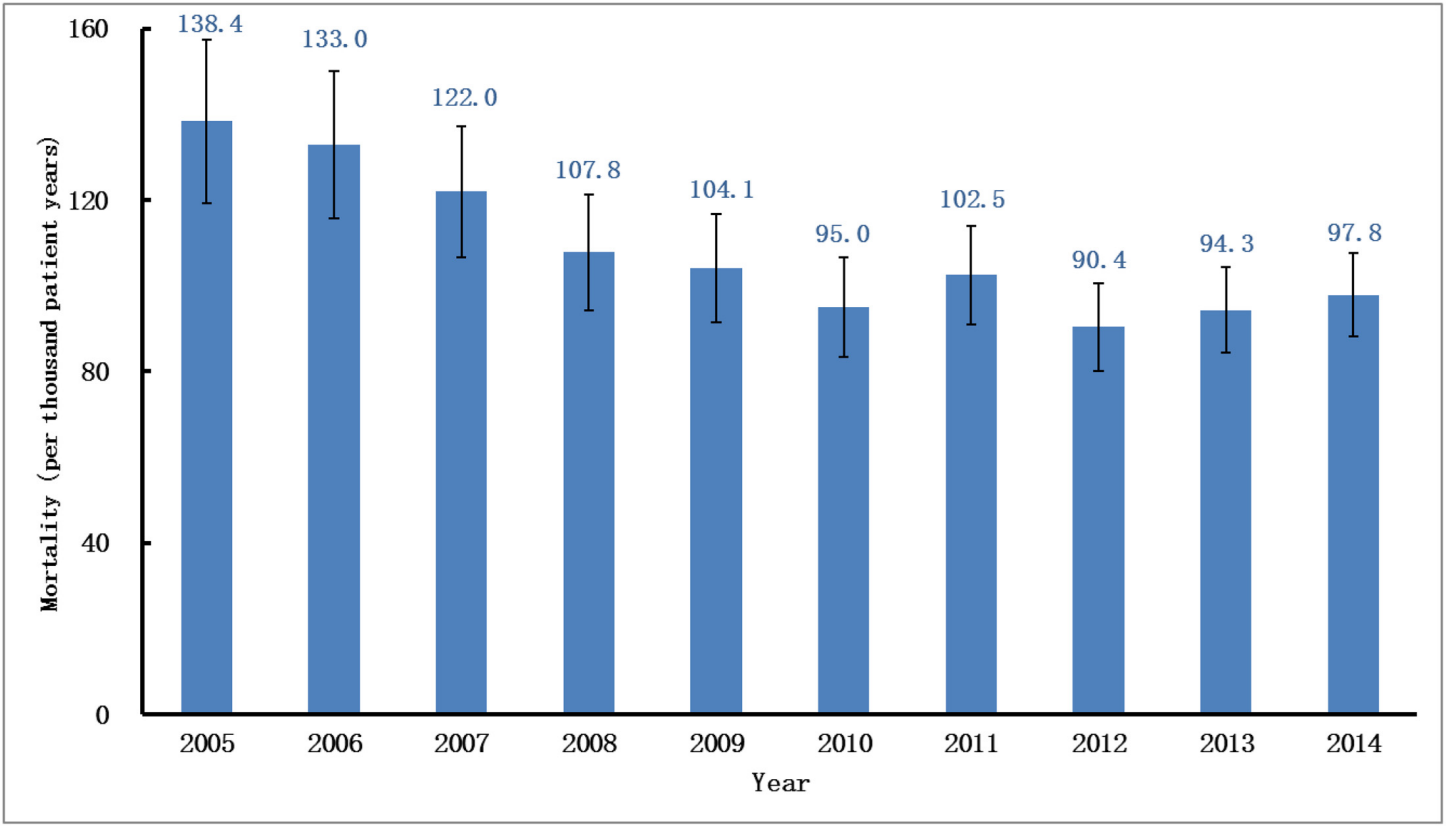
Annual mortality rate of RRT in UEBMI cohort. Mortality rates per 1000 patient-years at risk (adjusted for age and gender) over a ten-year period. RRT, renal replacement therapy; UEBMI, Urban Employee Basic Medical Insurance.

**Fig 8:**
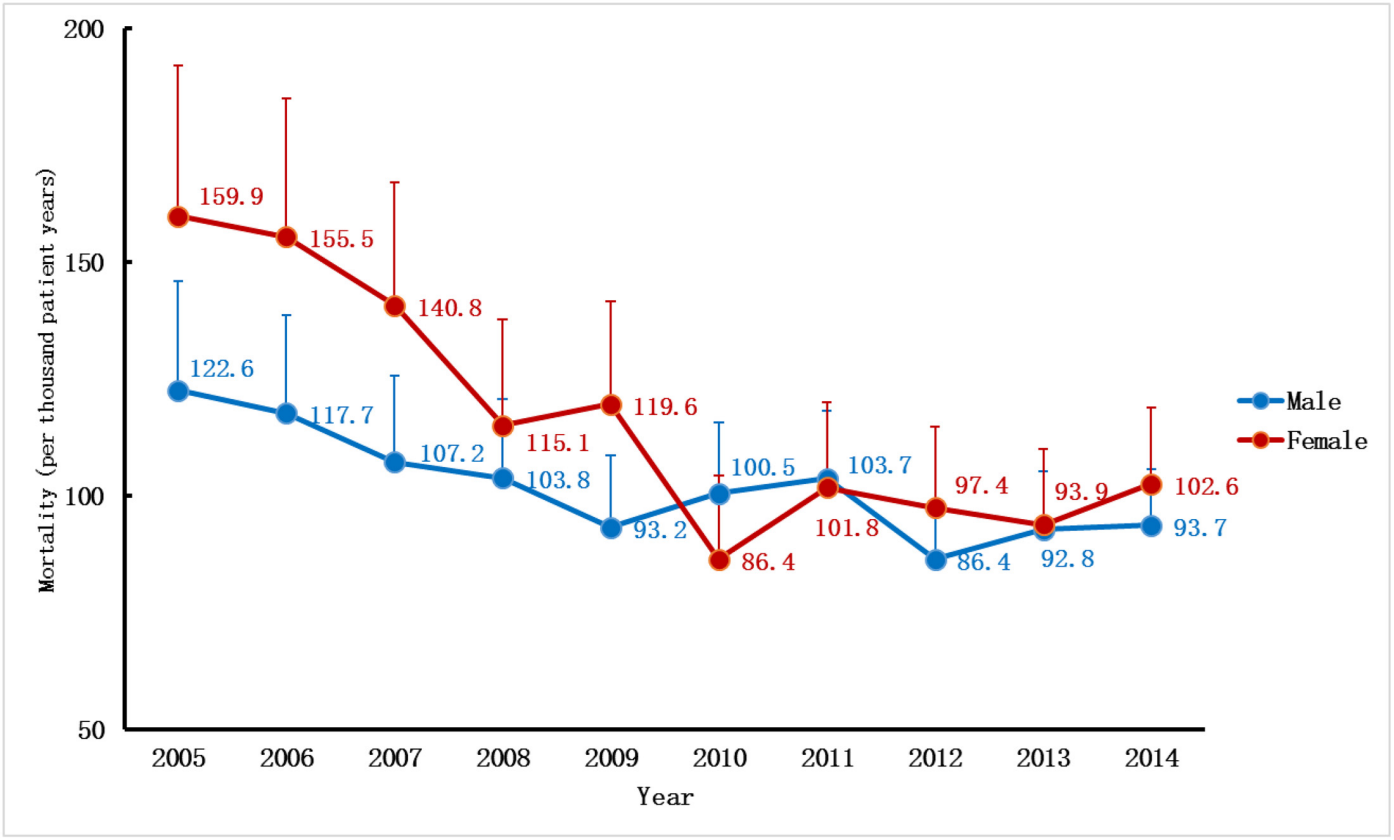
Gender-specific annual mortality rate of RRT in UEBMI cohort. Mortality rates per 1000 patient-years at risk by genders (adjusted for age) over a ten-year period. RRT, renal replacement therapy; UEBMI, Urban Employee Basic Medical Insurance.

The change trend in the annual mortality rate was similar to the incidence rate. From 2005 to 2010, the adjusted annual mortality rate of RRT-treated ESRD declined by 25% from 138.4 per 1000 patient-years to 95 per 1000 patient-years and came to a plateau in the sequential years (Fig 7, S3 File). The patterns of the mortality trends were also similar for both sexes. Regarding the adjusted gender-specific annual mortality rate, male subjects had consistently lower rates than female subjects, except for in 2010 and 2011. Over the past decade, the adjusted annual mortality rate for the male decreased by 24% from 122.6 in 2005 to 93.7 in 2014, while the rate for the female underwent a 36% decrease from 159.9 in 2005 to 102.6 in 2014 (Fig 8).

### Survival rate

**Fig 9:**
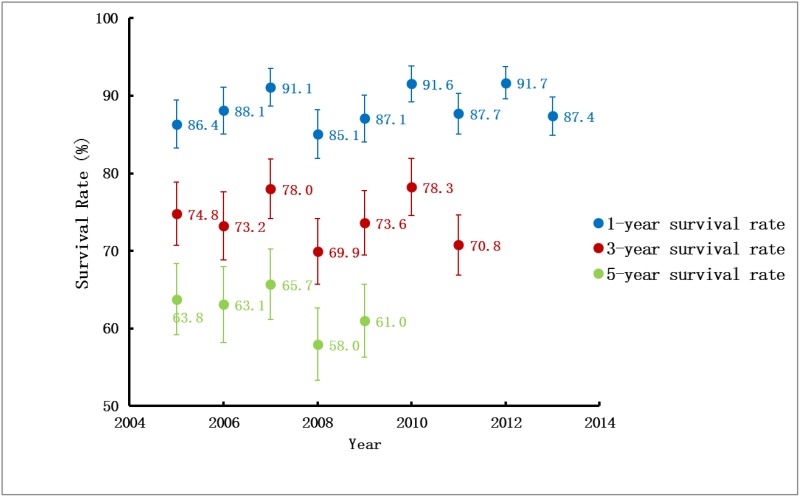
Change in long term survival rates of ESRD by year of starting RRT in UEBMI cohort. Unadjusted survival probability was calculated using the Kaplan-Meier methods and was expressed as percentage from 0 to 100. ESRD, End stage renal disease; RRT, Renal replacement therapy; UEBMI, Urban Employee Basic Medical Insurance.

The long-term survival rate of the RRT-treated ESRD fluctuated over the past ten years, with the 1-year survival rate ranging from 85.1% to 91.7%, the 3-year survival rate from 69.9% to 78.3% and the 5-year survival rate from 58% to 65.4% (Fig 9). Regarding the modality-specific long-term survival rate, kidney transplantation had a significant advantage over hemodialysis and peritoneal dialysis (both *P* < 0.01), whereas no significant differences were found between hemodialysis and peritoneal dialysis (*P* = 0.984). There were no significant differences between sexes (for peritoneal dialysis patients, *P* = 0.162; for transplantation patients, *P* = 0.335) except for hemodialysis patients, among whom the male had better survival rates than the female (for hemodialysis patients, *P* = 0.012).

## Discussion

As the world’s most populated developing country, China is facing a great challenge in its burden of NCD. Although the national prevalence of chronic kidney disease has been estimated at 10.8% [6], the exact prevalence and incidence of ESRD in China have not been well studied. By analysing Nanjing’s unique data system of RRT in UEBMI for ten years, we obtained for the first time the exact epidemiological data about RRT in urban employee in Nanjing, with the prevalence steadily increased to 1,228.6pmp (2014), and the incidence rate stabilized at approximately 220pmp (2014).

As illustrated in our study, the incidence of RRT declined to the plateau, reaching 218.8pmp in 2014 and the year 2009 was around the turning point. This trend of incidence was associated with the gradual process of the universal coverage of RRT in urban employee in Nanjing. Before the reimbursement program for RRT was introduced in 2000, RRT was not affordable for most ESRD patients. With its introduction, large influx of ESRD patients into the UEBMI cohort gradually diminished the treatment gap of RRT and this resulted in the abnormal phenomena of the falling trend of the high levels of RRT incidence during the early years. When universal coverage for ESRD in urban employee was achieved after 2009, the RRT incidence went to plateau and stabilized around its real level.

The prevalence of RRT steadily increased, rising from 891.7pmp in 2005 to 1,228.6pmp in 2014. This continuous increase was the result of the sustained improvement of the insurance program for ESRD patients in Nanjing besides the technical advancement. During this ten-year period, despite the large increase in total medical expenditures for ESRD patients, the percentage of the expenses covered by UEBMI increased from 77.8% in 2005 to 86.4% in 2014. Such sustainable improvements guaranteed the quality of treatment for ESRD patients and therefore improved their survival.

The falling trend of the mortality rate in the early years (2005–2010) might be associated with the large influx of the ESRD population right after the inception of the RRT reimbursement program. With the development of this program, the relative constant ESRD population and especially the improved insurance for RRT contribute to the stable mortality rate since 2010. The long-term survival rate of the RRT patients in the UEMBI cohort was comparable to that of their counterparts in developed countries [7–10]. Despite the improvement of insurance quality, the exclusion of the unemployed residents and the rural residents in our study may also raise the overall survival rate. As the excluded individuals were reported having worse prognosis [11, 12].

The burden of ESRD reflected from our study was the heaviest among all of the contemporary epidemiological studies of ESRD in China [13–19]. Among the limited ESRD data in China, two studies were based on nationwide ESRD survey conducted by the Chinese Society of Nephrology in 1999 and by the Chinese Society of Blood Purification in 2008. As reported in these two surveys, the nationwide prevalence rate of RRT increased from 33.16pmp in 1999 to 79.1pmp in 2008 and the nationwide incidence rate of RRT increased from 15.3pmp in 1999 to 36.1pmp in 2008. However, these two studies were facility-level surveys using survey forms, and the response rate was less than 50% in 1999 and was 87.0% in 2008. Due to lack of the strict nationwide electronic registry system based on individual data, the actual prevalence and incidence rates of RRT-treated ESRD in China were severely underestimated in those two surveys. Other studies were mainly based on the dialysis registry systems in Beijing and Shanghai. The prevalence and incidence rates of RRT in Beijing were reported as 579pmp and 94.4pmp, respectively, in 2013, while the prevalence and incidence rates of RRT in Shanghai were reported as 744.0pmp and 144.8pmp, respectively, in 2010. For these registry system based studies, the fact is that there was high mobility in the study populations, and patients received kidney transplantation were excluded from the studies in Shanghai, while only hemodialysis patients were included in Beijing studies excluding patients with kidney transplantation and peritoneal dialysis, suggesting that all these reports on epidemiology of RRT-treated ESRD were actually based on an incomplete data system. Despite the incompleteness of the dialysis registry systems, the enormous treatment gaps in the past may also contribute to the large differences in the reported ESRD data in China.

As the world’s most populated developing country, the disparities in regional economies and levels of medical care were considerable, and treatment gaps for ESRD remained large in undeveloped regions in China. In 2009, the central government of China launched an ambitious health care reform and directed subsidies towards those undeveloped regions. In 2012, RRT for ESRD was included in the reimbursement program of the basic medical insurance. With the increase in government-directed health care reform in China, universal coverage of medical insurance will eventually be achieved for all 1.3 billion people, and RRT will eventually be accessible and affordable for all ESRD patients [20]. Based on the ten-year experience from Nanjing, the projected number of ESRD patients requiring RRT in China will experience an explosive increase in the next decade. The government, medical communities and especially nephrology specialists should be well prepared and start to take active measures to control this coming crisis.

Some limitations should be carefully considered. Only the UEBMI database was used for our analysis, while individuals insured by URBMI or NCMS and the uninsured were not included. In analysis, ESRD patients with incomplete information (16subjetcs) were also excluded, given their small percentage, the impact could be omitted. Urban resident without employment, the rural resident and the uninsured thus formed the uncovered portion in our study. These individuals had lower incomes, weaker health awareness and their medical insurance was lower in levels. Accordingly, they were less likely to get adequate intervention on hypertension, diabetes and chronic kidney disease before the development of ESRD. As demonstrated in studies using USRDS database, these individuals had a higher incidence of ESRD and had a lower long-term survival rate after RRT [11, 12]. And the exclusion of these individuals in our study constituted certain selection bias. The real burden of ESRD in Nanjing in the past decade could be even higher and their long-term survival might not be so optimistic. Also, as our study population was confined to the insured urban population in developed area in China, extrapolation to other types of populations in China should be made with caution. More data is needed from larger population based on complete registry systems to gain a better insight into the ESRD epidemiology in China.

In summary, our study based on the ten-year complete registry data on RRT in urban employees in Nanjing suggested an increasing prevalence of ESRD patients in Chinese population. This provided us a unique opportunity to understand the real threat of ESRD confronting China during its process of health care transition.

## Supporting Information

S1 Fig RRT modality ratio of prevalent ESRD patients in UEBMI cohort. The ratio for each RRT modality was calculated as the percentage of the prevalent ESRD population in the UEBMI cohort. RRT, renal replacement therapy; ESRD, End stage renal disease; HD, hemodialysis; PD peritoneal dialysis; Tx, kidney transplantation.
(TIF)

S2 Fig RRT modality ratio of incident ESRD patients in UEBMI cohort. The ratio for each RRT modality was calculated as the percentage of the incident ESRD population in the UEBMI cohort. RRT, renal replacement therapy; ESRD, End stage renal disease; HD, hemodialysis; PD peritoneal dialysis; Tx, preemptive kidney transplantation.
(TIF)

S1 File Modality-specific data for incidence, prevalence, mortality and long-term survival rate. (XLS)

S2 File 95% confidence intervals for data presented in each figure. (XLS)

S3 File Data of piecewise regression. (XLS)
