# Supplementary figures and images for: Epidemiological Study of RRT-Treated ESRD in Nanjing - A Ten-Year Experience in Nearly Three Million Insurance Covered Population
Source: PLoS One. 2016 Feb 18;11(2):e0149038. doi: 10.1371/journal.pone.0149038 (PMC4758634; doi:10.1371/journal.pone.0149038)

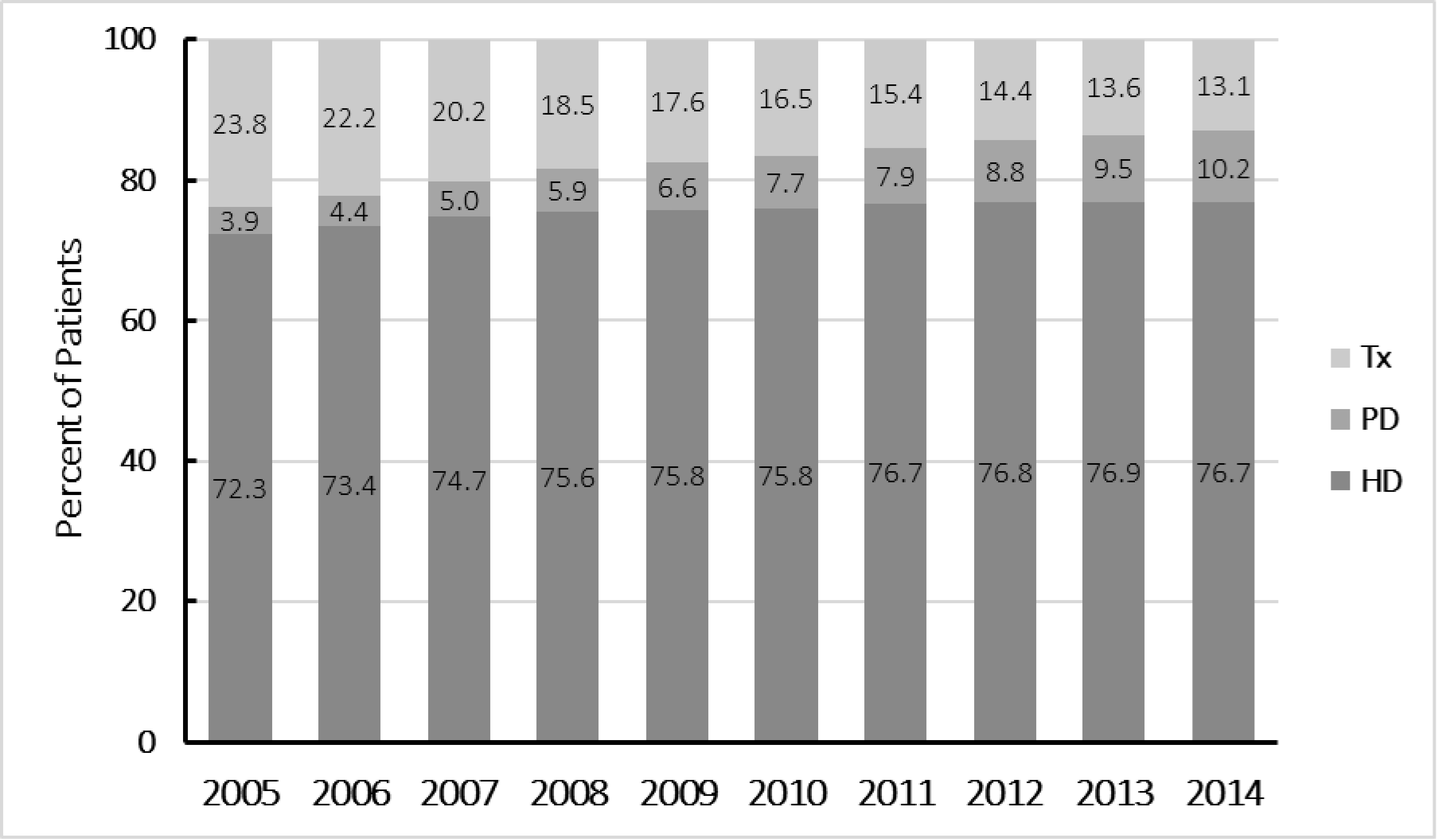

Supplement: S1 Fig — The ratio for each RRT modality was calculated as the percentage of the prevalent ESRD population in the UEBMI cohort. RRT, renal replacement therapy; ESRD, End stage renal disease; HD, hemodialysis; PD peritoneal dialysis; Tx, kidney transplantation. (TIF) [file pone.0149038.s001.tif]

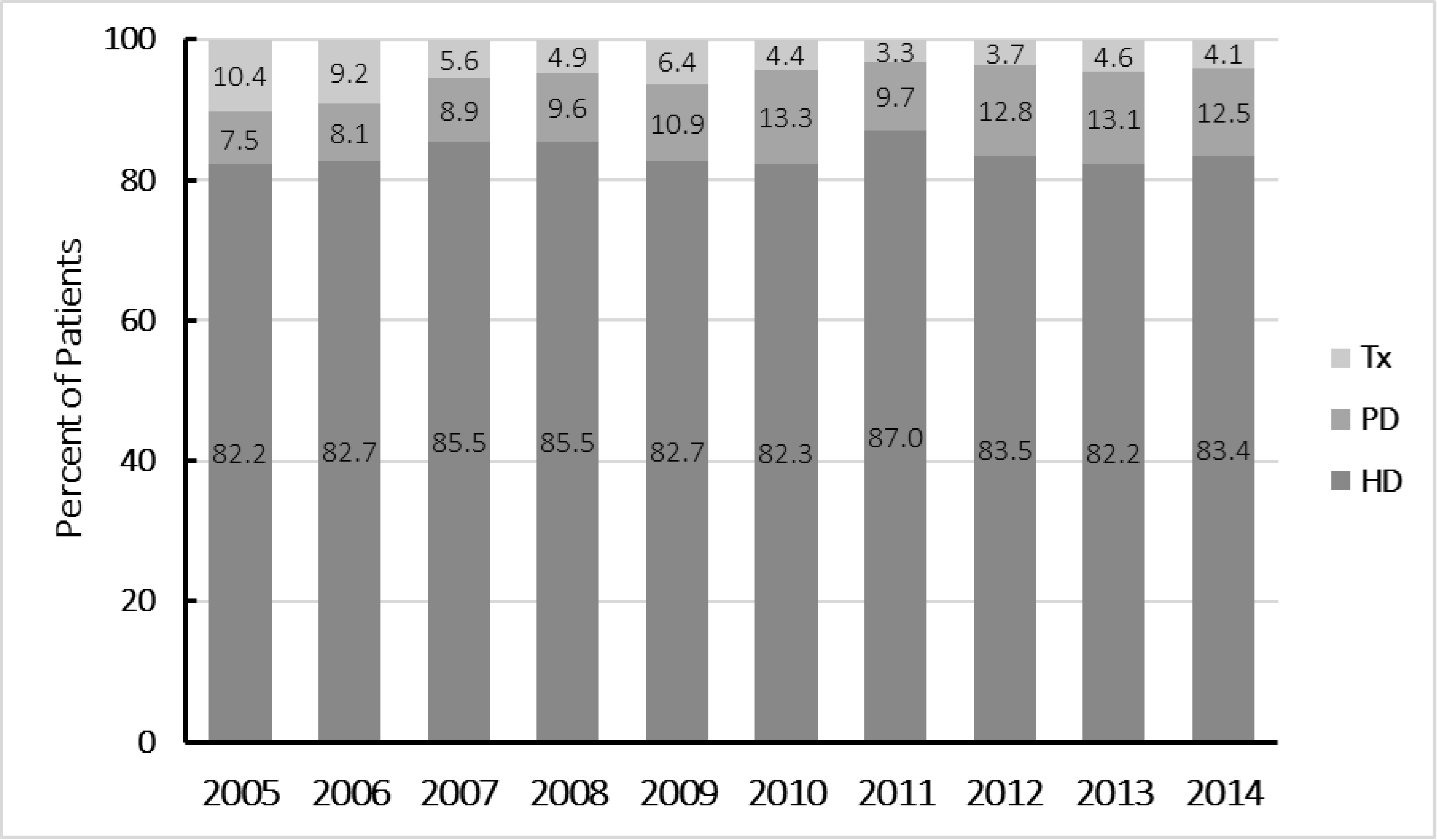

Supplement: S2 Fig — The ratio for each RRT modality was calculated as the percentage of the incident ESRD population in the UEBMI cohort. RRT, renal replacement therapy; ESRD, End stage renal disease; HD, hemodialysis; PD peritoneal dialysis; Tx, preemptive kidney transplantation. (TIF) [file pone.0149038.s002.tif]
